# The current state of the genetic counselor profession in the German-speaking countries

**DOI:** 10.1016/j.gimo.2024.101849

**Published:** 2024-05-15

**Authors:** Gunda Schwaninger, Simone Heidemann, Sabine Rudnik-Schöneborn, Johannes Zschocke

**Affiliations:** 1 Institute of Human Genetics, Medical University of Innsbruck, Innsbruck, Austria; 2 Institute of Tumor Genetics North, Kiel, Germany

**Keywords:** European Board of Medical Genetics (EBMC), German Society for Human Genetics (GfH), MSc in Genetic and Genomic Counseling (GGC-MSc), Professional Association of German Geneticists, Berufsverband der Deutschen Humangenetiker eV, BVDH

## Introduction

Until recently, Germany, Austria, and the German-speaking part of Switzerland lacked genetic counselors (GCs). However, in 2019, the Medical University of Innsbruck initiated an MSc program in Genetic and Genomic Counseling (GGC-MSc), igniting the introduction of the profession in the region. This article aims to present the current state of the GC profession in the area and highlights the efforts made to address various challenges in integrating GCs into genetic services.

### Educating GCs in Germany, Austria, and Switzerland (the D-A-CH region)

The first group of 7 students from Germany and Austria successfully completed the program in 2022. The second group of 9 students from the D-A-CH region, is graduating in the spring of 2024. In September 2023, the third cohort, consisting also of 9 students, enrolled. All current students and graduates are actively engaged in human/medical genetic centers within the D-A-CH region. Prospective applicants from diverse professional backgrounds are encouraged to apply to the MSc program. The universities’ selection process requires that they possess a minimum of a bachelor’s degree in a biomedical science, psychology, or a related academic health care discipline, coupled with at least 2 years of relevant work experience. Among the 25 students and graduates so far, 11 held doctoral degrees, 7 possessed master’s degrees, and 7 had bachelor’s degrees upon enrollment (Table 1). Given their prior engagement in genetics, most students started with a robust understanding of the field. Many applicants transition within genetic centers, having previously served in roles such as clinical laboratory geneticists, biomedical laboratory technicians, or in administrative capacities within clinical settings. This background offers several advantages for this regionally new profession. Employers actively support role transitions within the workplace, fostering trust and backing for the GC profession. The often long-standing personal and professional relationships between GC trainees and their clinical mentors are a great advantage and may partly explain the high prior qualifications of many students far beyond the entry requirements of the program. The diverse composition of the student body enriches the teaching environment and fosters an inspiring and collaborative atmosphere. Students are encouraged to combine the 5 semesters of the GGC-MSc program with part-time employment in a genetic service, enabling a balanced approach to handling teaching sessions, assessments, clinical training, work, and personal commitments.

**Table 1:** Academic qualifications and country of residence of students in the Innsbruck MSc program in Genetic and Genomic Counseling

Student Intake	Qualification Before Entering GGC-MSc Program			Country of Residence		
	PhD/equivalent	Master/equivalent	BSc/equivalent	Germany	Austria	Switzerland
1st student cohort (2019-2022)	3	1	3	6	1	-
2nd student cohort (2021-2024)	3	5	1	5	2	2
3rd student cohort (2023-2026)	5	1	3	6	1	2
Total 25 students	11	7	7	17	4	4

We hope to see more academic GC programs in the German-speaking countries in the coming years to further increase the number of trained GCs. Major challenges are the unfamiliarity with the profession both in the public and the professional realm, as well as the small number of senior GCs to train students. We therefore foster international collaborations with experienced GCs in teaching and clinical training of our GC students. Also, standardization of both education and GC competencies needs to be a major focus as the GC profession expands.

### Registration board for GCs

The European Board of Medical Genetics (EBMG) serves as a professional entity upholding professional standards in the education, training, and practice of European GCs, clinical laboratory geneticists, and medical geneticists.^1^, ^2^, ^3^, ^4^ When designing the GGC-MSc program curriculum in Innsbruck, it was crucial to offer students an internationally accredited degree and the option for a European registration as the GC profession is not a recognized profession in most of the D-A-CH region so far. In 2021, the EBMG officially accredited the GGC-MSc program at the Medical University of Innsbruck, paving the way for graduates to attain registration as European certified GCs.^3^^,^^5^ The European curriculum and the international recognition enables GCs to practice their profession across a range of European nations, including the UK and France, where the GC profession is well established.^6^, ^7^, ^8^ The process for GC registration entails a minimum of 8 years. To enter a GC program, candidates must hold a relevant bachelor’s degree or equivalent. Upon successful completion of the GC master’s degree, which extends over a period of 2 to 3 years, graduates then engage in a 2-year, full-time trainee phase within a clinical genetic service under the guidance of medical geneticists. After this training phase, individuals compile a registration portfolio for submission to the EBMG, comprising a case log demonstrating a minimum of 50 fully managed consultations across diverse cases, 2 case studies, evidence of continuing education, clinical and counseling supervision, and 2 references.^4^ EBMG registration has to be renewed every 5 years.

Currently, only 4 more senior GCs are employed in the German-speaking area: 2 in Germany, 1 in Switzerland, and 1 in Austria. All of them were trained abroad (United Kingdom, Spain, India, and United States), and some have many years GC work experience in other countries and only moved to the region in the last few years. To compensate this shortage of senior GCs in the region, efforts are made to expose students to diverse counseling approaches by both medical geneticists in the D-A-CH region and GCs in French-speaking Switzerland and the United Kingdom during their clinical training.

### Professional organizations for GCs in the D-A-CH region

The German Society for Human Genetics (GfH) and the Professional Association of German Geneticists (Berufsverband der Deutschen Humangenetiker eV, BVDH) have recognized the need to expand the currently limited capacities for genetic counseling in Germany, and some geneticists explicitly proposed the introduction of nonphysician GCs.^9^, ^10^, ^11^ After intense discussions at the GfH annual conference in 2019 about the introduction of GCs, a growing consensus gradually emerged. In 2019 the GfH and BVDH jointly applied to the German ministry of health for the official recognition of GCs and clinical laboratory geneticists.^12^ At the annual GfH conference in 2022, the GfH and the BVDH officially announced the implementation of GCs as one of their major strategic goals. Presently, ongoing negotiations are taking place between the already qualified German-speaking GCs and the GfH and BVDH on the most effective means of integrating GCs into the respective professional bodies. Everyone involved agrees that integration and mutual collaboration within the GfH and BVDH is preferable to a model in which GCs form a separate association. A structure akin to the European Society of Human Genetics is envisioned, in which medical geneticists, clinical laboratory geneticists, and GCs constitute the different branches of the EBMG and function within a multiprofessional genetics team.^2^ Achieving this goal will require time and a mutually agreeable and respectful approach. First of all, a professional title for GCs that presents an equivalent German translation, acceptable to both medical geneticists and GCs has to be agreed on. Currently, the term “Genetische:r Fachberater:in”—in correspondence to the Fachhumangenetiker:in (clinical laboratory geneticist) and Facharzt/Fachärztin (medical specialist)—is under consideration. Additionally, there are negotiations under way to establish a commission within the GfH dedicated to address the numerous challenges faced by the GC profession, encompassing issues such as recognition, scope of practice, and regulation. Remuneration and billing in Germany fall primarily into the competence of the BVDH. Furthermore, there is the proposal to create an interest group for GCs—a larger, more loosely structured network—to advocate for and support the introduction of the profession within and beyond the professional bodies. Because of their small number, the Austrian and Swiss GCs have decided to join these collective efforts, aiming at a harmonized recognition across all 3 German-speaking countries.

A recent report from the Federal Commission for Genetic Testing in Humans in Switzerland further underscores the increasing demand for health care professionals capable of delivering genetic counseling.^13^ Despite the absence of fully trained German-speaking GCs in Switzerland thus far, the French-speaking region has had GCs trained in France and the United Kingdom for years. The first 11 Swiss French-speaking GCs established the Association Suisse des Conseillers en Génétique in 2016, advocating for professional recognition and the complete acknowledgment of GC competencies within Switzerland. A first milestone was achieved in 2008 in one of the 26 Swiss provinces (17 German speaking), the Canton of Vaud/Waadt, which instituted the first official regulations for GCs. Recently, the Swiss Federal Commission for Genetic Testing in Humans appointed 2 GCs to their board, signifying an emerging recognition of the pivotal role of GCs in the field. Encouragingly, German-speaking GCs are welcomed to participate in both the French- and German-speaking professional societies, fostering ongoing exchange and collaboration between GCs from both linguistic groups.

### Scope of practice and competency standards

One major challenge of introducing a new health care profession lies in the absence of a clearly defined role for GCs within interprofessional genetics teams in the D-A-CH region. Local GCs are striving to engage in the internationally defined core activities for GCs, integral to their MSc education.^14^ Beyond that, there is a critical need to establish a precise definition of the scope of practice that aligns with both international competency standards, as well as the legal frameworks in the German-speaking countries. When referring to international documents the scope of practice is defined for countries in which the GC profession is well established but mostly unavailable for countries in which the profession is only emerging. A comprehensive list detailing the professional roles for medical geneticists, GCs, and genetic counseling assistants/genomic associates (without a GC master’s degree) was recently published in the United Kingdom and more valuable documentation on the scope is available for Australasia.^15^^,^^16^ Both can be largely applied within the D-A-CH region. The recent study of Ormond et al^17^ investigated the most commonly performed GC tasks, surveying 189 GCs from 22 countries. The activities most routinely undertaken by surveyed GCs included case preparation, contracting, establishing rapport, family and medical history taking, risk assessment, and counseling. These activities provided education on basic genetic information, test options, outcomes, and implications, including management recommendations based on test results. Additionally, facilitating informed decision making and assessing patients’ psychosocial needs and background to recognize factors impacting counseling interaction were common tasks.^17^ Conversely, tasks within the medical history category, such as physical examinations, were among the least performed.^17^ This corresponds to interview studies and workshops conducted with medical geneticists in the D-A-CH region, defining physical examinations, syndromology, genetic test orders, and the diagnostic interpretation of the relationship of variants and clinical phenotype as medical tasks. However, they acknowledged GCs’ capabilities in most other aspects of a genetic consultation, contingent upon their experiences.^13^^,^^14^^,^^18^^,^^19^ The GC commission proposed to be established within the GfH, faces the crucial challenge of outlining a clear definition of the GC scope of practice, beginning from training levels to the trainee phase and extending to EBMG registered GCs. It is imperative to recognize that GCs are academically trained health care professionals following international competency standards and not administrative assistants.

### Regulation

Graduates from EBMG accredited MSc programs in genetic counseling undergo training aligned with a European core curriculum, enabling their entry into the European registration system for GCs. However, the scope of practice for GCs varies according to national regulations. Understanding what GCs are permitted to do within specific countries is pivotal. Switzerland recently revised its genetics law,^20^ mandating that a physician ordering a genetic analysis must provide or arrange genetic counseling. In this context, genetic counseling can be conducted by any “competent person” (“fachkundige Person”) not explicitly categorized as a physician. Nonetheless, the authorization to order genetic analyses is reserved for medical geneticists or specialized physicians with additional genetics training (§ 21^2^ Genetische Untersuchungen beim Menschen).

The German law—Gendiagnostikgesetz—allows nonphysician experts to participate in genetic counseling with the consent of the individual concerned (§ 10^3^ Gendiagnostikgesetz). The critical aspect involves the delegation of medical tasks associated with genetic consultations, which is not specifically outlined in the “Bundesmantelvertrag 2023” for physicians (federal treaty on insurance coverage). Legally, only tasks outside a physician’s specific competence can be delegated and subsequently reimbursed. However, interpretation of what precisely falls under this scope remains subjective. GCs can assume various delegable tasks; yet, the patient must sign the informed consent for genetic analyses in the presence of the supervising physician.

Similarly, the Austrian law, the Gentechnikgesetz, necessitates a genetic consultation to be performed by a genetically trained medical specialist. However, in relevant circumstances, additional nonmedical counseling from a psychologist/psychotherapist or social worker must be provided (§ 69^4^ Gentechnikgesetz).^21^ Despite the fact that the law does not consider the GC profession, it explicitly indicates the possibility and sometimes the necessity to involve other competent professionals in genetic consultations.

### Remuneration

The financial compensation of GCs holds significant importance for employees and employers. It is crucial for genetic institutions to devise viable funding strategies for new positions and to establish appropriate levels of remuneration. As GC students join the MSc program with diverse prerequisites, their salaries hinge on various factors such as prior qualifications, academic achievements, professional background, and job responsibilities. In general, a significant advantage of GCs lies in their tendency to remain within a service for an extended period, thereby making the time provided for their training a valuable long-term investment. Typically, a GC holding a master’s degree can anticipate placement on a salary scale between that of a laboratory technician and a clinical laboratory geneticist. Some medical geneticists have also proposed equal compensation for GCs and medical geneticists in training.^14^ Considering the extensive education and experience, a registered GC with a PhD should ideally receive compensation on par with a clinical laboratory geneticist.^14^

### Billing

Unresolved issues persist regarding the billing of GC services in the D-A-CH region. Presently, the German Bundesmantelvertrag does not delineate the delegability of medical genetic counseling services,^22^ and the Einheitlicher Bewertungsmaßstab 2024 (German evaluation standard for medical genetic tasks) lacks a service code for GCs.^23^ Although academic institutions have the option to employ GCs as part of their scientific staff, this poses a significant challenge for genetic services outside universities and hospitals. In the context of these services, physicians face limitations in billing patients based on their allocated working time, adhering strictly to plausibility guidelines. This structure does not accommodate billing for additional time spent by GCs with patients, potentially hindering the translation of growing capacities resulting from GC involvement into an increase in billed hours. Consequently, there is a pressing need for updated billing systems. One potential solution is the introduction of a unified billing system applicable to both physicians and GCs, contingent upon a clear definition of what tasks medical geneticists can delegate to GCs.

In accordance with the enormous effort in other countries, we will work toward the listed challenges, including the scope, regulation, remuneration, and billing of GCs, in reference to regions in which the GC professional development is more advanced.^15^^,^^16^ Over the coming years we strive to establish the GC profession in close collaboration with the members of the medical genetics team. One next step is the establishment of a GC commission within the German Society of Human Genetics.

### Conclusion

As a new cohort of master trained GCs graduates every 2 years from the Medical University of Innsbruck, the profession is gradually evolving. Patient acceptance is high, and the direct correlation between job satisfaction and the integration of GCs as valued members within interprofessional teams is evident. To attract professionals to this emerging field, it is essential to offer them clarity regarding their scope of practice, anticipated salaries, and the regulatory framework governing their occupation. Establishing the GC profession in the D-A-CH region necessitates providing new GCs and their prospective employers with reassurance concerning the creation of GC positions. Moving forward, efforts should also concentrate on negotiating billing strategies with insurance companies—a task that depends on the collaborative efforts of medical geneticists and GCs. Presently, the initial group of approximately 20 GCs is dedicated to improving close collaboration with the D-A-CH societies and professional associations of human genetics.

## Data Availability

The manuscript is mainly a portrait of the current state of the profession, and details are either cited or respond to knowledge pertained as the program director of the first MSc in Genetic and Genomic Counseling. All data are contained in the manuscript.

## Declaration of AI and AI-Assisted Technologies in the Writing Process

During the preparation of this work the first author used ChatGPT in to check for language, spelling, and grammar. After using this tool, the first author reviewed and edited the content as needed and takes full responsibility for the content of the publication.

## Conflict of Interest

Author Simone Heidemann is a graduate of the MSc program in Genetic and Genomic Counseling at the Medical University of Innsbruck. Authors Gunda Schwaninger, Sabine Rudnik-Schöneborn, and Johannes Zschocke are program director and part of the development team of the master program in Genetic and Genomic Counseling at the Medical University of Innsbruck.
